# Supplementary material for: Cumulative Weighing of Time in Intertemporal Tradeoffs
Source: J Exp Psychol Gen. 2016 Sep;145(9):1177–205. doi: 10.1037/xge0000198 (PMC4998108; doi:10.1037/xge0000198)
Supplement: Supplementary file 1 [file Scholten_Individuals.zip › plots/e30_p414_eg2_priors_and_posteriors.pdf]

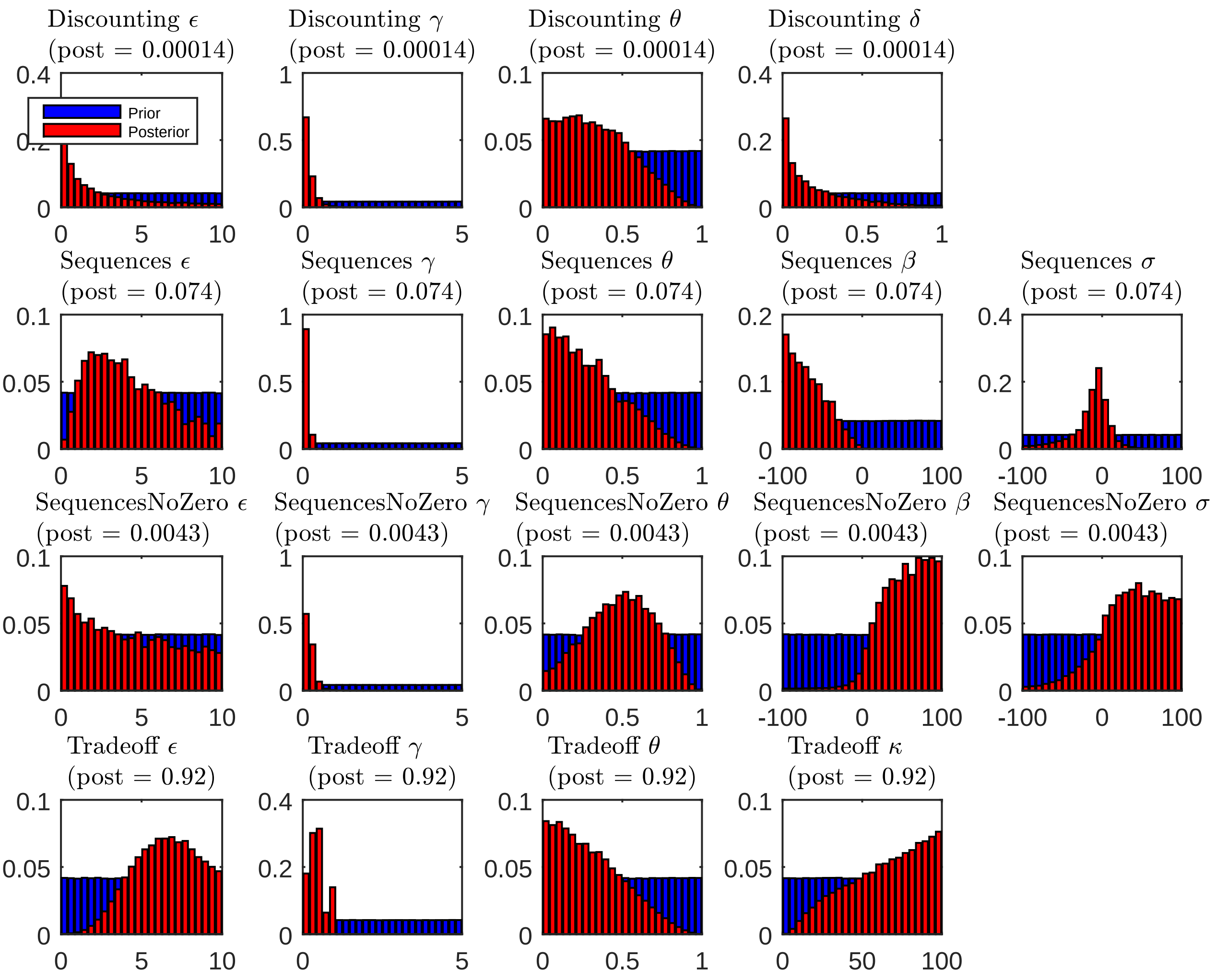
Discounting ε (post = 0.00014)
Discounting γ (post = 0.00014)
Discounting θ (post = 0.00014)
Discounting δ (post = 0.00014)
Prior
Posterior
Sequences ε (post = 0.074)
Sequences γ (post = 0.074)
Sequences θ (post = 0.074)
Sequences β (post = 0.074)
Sequences σ (post = 0.074)
SequencesNoZero ε (post = 0.0043)
SequencesNoZero γ (post = 0.0043)
SequencesNoZero θ (post = 0.0043)
SequencesNoZero β (post = 0.0043)
SequencesNoZero σ (post = 0.0043)
Tradeoff ε (post = 0.92)
Tradeoff γ (post = 0.92)
Tradeoff θ (post = 0.92)
Tradeoff κ (post = 0.92)
0
0.05
0.1
0.2
0.4
0.5
1
5
10
50
100
-100
